# An Account of Diseases in an Eastern District of London, from March 20 to April 20, 1810

**Published:** 1810-05

**Authors:** 


### *An Account of Diseases in an Eastern District of London, from March 20 to April 20, 1810.*

| ACUTE DISEASES. | |
|---|---|
| Peripneumonia Notha | 3 |
| Febris Lenta | 2 |
| Hæmoptysis | 4 |
| Catarrhus | 6 |
| Rheumatismus Acutus | 3 |

| CHRONIC DISEASES. | |
|---|---|
| Tussis | 15 |
| Tussis cum Dyspnœa | 13 |
| Phthisis Pulmonalis | 2 |
| Pleurodyne | 3 |
| Hæmoptysis | 5 |
| Hysteria | 4 |
| Cephalæa | 7 |
| Paralysis | 3 |
| Obstipatio | 5 |
| Hernia | 1 |
| Hæmorrhois | 1 |
| Rheumatismus Chronicus | 12 |

| INFANTILE DISEASES. | |
|---|---|
| Aphthæ | 3 |
| Ophthalmia | 2 |
| Diarrhœa | 5 |
| Vermes | 4 |
| Scrophula | 4 |

| PUERPERAL DISEASES. | |
|---|---|
| Menorrhagia Lochialis | 3 |
| Dolores Post Partum | 4 |
| Dysuria | 5 |
| Hæmorrhois | 3 |
| Mastodynia | 4 |

The long continuance of the cold weather, with the prevalence of East and North-East winds, has protracted the duration of those complaints which occur principally in the winter season.

We have therefore upon our list a large number of the same diseases as those which we have for some time announced. The connection of hæmoptysis with other affections of the lungs, and which we hinted at in a former report, still continues. In some of those cases, in which it before occurred, it has returned with increased violence; and in addition to these, fresh instances have appeared. This symptom appeared in an elderly subject during an attack of peripneumonia notha, and has returned at intervals for a considerable time since the termination of the original disease.

Rheumatic affections have lately appeared in a variety of forms. These might indeed be expected as the consequence of the late variable weather. The bright appearance of some mornings would naturally induce even valetudinarians to quit their home, and to enjoy the freshness of the air, and the beauty of the scene, inattentive to the inconvenience they might feel from the cool temperature of the atmosphere around them.

*Meteoro-*

